# How spatio-temporal habitat connectivity affects amphibian genetic structure

**DOI:** 10.3389/fgene.2015.00275

**Published:** 2015-09-08

**Authors:** Alexander G. Watts, Peter E. Schlichting, Shawn M. Billerman, Brett R. Jesmer, Steven Micheletti, Marie-Josée Fortin, W. Chris Funk, Paul Hapeman, Erin Muths, Melanie A. Murphy

**Affiliations:** ^1^Department of Ecology & Evolutionary Biology, University of TorontoToronto, ON, Canada; ^2^Department of Natural Resources Management, Texas Tech UniversityLubbock, TX, USA; ^3^Department of Zoology and Physiology, University of WyomingLaramie, WY, USA; ^4^Program in Ecology, University of WyomingLaramie, WY, USA; ^5^School of Biological Sciences, Washington State UniversityPullman, WA, USA; ^6^Graduate Degree Program in Ecology, Department of Biology, Colorado State UniversityFort Collins, CO, USA; ^7^Department of Biology, Central Connecticut State UniversityNew Britain, CT, USA; ^8^Fort Collins Science Center, U.S. Geological SurveyFort Collins, CO, USA; ^9^Department of Ecosystem Science and Management, University of WyomingLaramie, WY, USA

**Keywords:** boreal chorus frog (*Pseudacris maculata*), functional connectivity, dispersal, gravity model, landscape genetics, metapopulation dynamics, spatio-temporal dynamics

## Abstract

Heterogeneous landscapes and fluctuating environmental conditions can affect species dispersal, population genetics, and genetic structure, yet understanding how biotic and abiotic factors affect population dynamics in a fluctuating environment is critical for species management. We evaluated how spatio-temporal habitat connectivity influences dispersal and genetic structure in a population of boreal chorus frogs (*Pseudacris maculata*) using a landscape genetics approach. We developed gravity models to assess the contribution of various factors to the observed genetic distance as a measure of functional connectivity. We selected (a) wetland (within-site) and (b) landscape matrix (between-site) characteristics; and (c) wetland connectivity metrics using a unique methodology. Specifically, we developed three networks that quantify wetland connectivity based on: (i) *P. maculata* dispersal ability, (ii) temporal variation in wetland quality, and (iii) contribution of wetland stepping-stones to frog dispersal. We examined 18 wetlands in Colorado, and quantified 12 microsatellite loci from 322 individual frogs. We found that genetic connectivity was related to topographic complexity, within- and between-wetland differences in moisture, and wetland functional connectivity as contributed by stepping-stone wetlands. Our results highlight the role that dynamic environmental factors have on dispersal-limited species and illustrate how complex asynchronous interactions contribute to the structure of spatially-explicit metapopulations.

## Introduction

A fundamental goal of ecology is to understand how environmental variation influences species persistence, abundance, and gene flow (Cushman, 2006; Gomez-Rodriguez et al., 2009; Goldberg and Waits, 2010). Landscape heterogeneity is defined by fluctuations in environmental conditions that range from relatively invariable (e.g., topography, soil texture) to highly variable (e.g., rainfall, vegetation abundance) over multiple spatial and temporal scales. For many species, these abiotic conditions are necessary for species survival, recruitment, (Fahrig, 2003; Ewers and Didham, 2007), and dispersal (Girdner and Larson, 1995; Driscoll, 1997; Schwartz and Jenkins, 2000; Banks et al., 2004; Mokany, 2007). Yet it remains unclear to what extent variation in suitable conditions over space and time affects gene flow, population genetic structure, and genetic diversity of natural populations.

Functional connectivity, the degree to which the environment impedes or facilitates the movement of individuals among resource patches (Taylor et al., 1993; Bélise, 2005), is linked to genetic connectivity between populations existing in spatially-explicit habitat patches (Brown and Kodric-Brown, 1977; Tallmon et al., 2004). Geographic distance is expected to play a significant role in the explanation of genetic distance between a pair of occupied sites (McRae, 2006), assuming dispersal is limited over large distances. Yet the functional connectivity of a species may be dependent on environmental characteristics within- and between-habitat patches, whereby landscape condition may create resistance to gene flow in addition to animal movement.

Within-habitat characteristics (e.g., vegetation, resource abundance, presence of conspecifics) affect dispersal by influencing the production and survival of migrants (Banks et al., 2004), while between-habitat patch factors (e.g., inter-patch matrix: complex topography, vegetative cover, risk of predation) affect the probability of colonization and establishment (Stow and Sunnucks, 2004) in destination habitat patches. Temporal fluctuations in these environmental characteristics may mediate the complex ecological interactions that influence demographic and genetic processes within and between natural populations (Gomez-Rodriguez et al., 2009; Velo-Antón et al., 2013), especially for dispersal-limited species. It is therefore expected that fluctuating, heterogeneous landscapes will affect species functional connectivity corresponding to either beneficial or detrimental effects on demographic and dispersal thresholds essential for species persistence and genetic diversity (Schwartz and Jenkins, 2000; Scherer et al., 2012). Quantified values of functional connectivity can help characterize complex spatio-temporal interactions between landscape composition and configuration, population genetic structure, and genetic connectivity of a population.

Amphibians are exemplary model species to assess genetic connectivity in spatially and temporally variable landscapes because they are dispersal-limited, patch-dependent species (Gamble et al., 2007) sensitive to changes in vegetation and fluctuating hydrologic conditions. Juveniles leave ephemeral wetlands after metamorphosis, usually as wetlands are drying (Semlitsch, 2008) and are then subject to the spatio-temporal dynamics of the within-patch matrix. Successful recolonization of wetland habitat patches is more likely between neighboring patches than distant, isolated patches (Driscoll, 1997; Smith and Green, 2005; Rozenfeld et al., 2008) especially if the between-patch matrix is resistant to movement. Within- and between-patch environmental fluctuations may significantly influence amphibian occupancy of surrounding wetland patches (Scherer et al., 2012) altering amphibian population dynamics and genetic structure. However, these interactions between variable abiotic conditions and amphibian population genetic structure are understood poorly despite significant implications for population persistence, species diversity, and metapopulation dynamics as the environment changes and the climate warms. Thus, our goal was to test the effects of fluctuating environmental factors on functional connectivity of an amphibian population using a landscape genetics approach.

We evaluated the effect of spatio-temporal variation in wetland availability on the genetic connectivity of a population of boreal chorus frogs (*Pseudacris maculata*) in the high mountain wetlands of the Northern Rocky Mountains in Larimer County Colorado. Across the species range, boreal chorus frogs breed in primarily ephemeral wetlands with emergent vegetation in spring and summer. Individuals then disperse to wet meadows to forage during the summer and early fall (Weyrauch and Grubb, 2004). Spencer (1964) described the species' relatively low dispersal ability (~600 m average dispersal maxima) and variation in site occupancy within years across our study area. Given pond-breeding behavior and low dispersal capacity, boreal chorus frogs are well-suited for measuring environmental limits to genetic connectivity. Moreover, snowpack has the potential to have a direct effect on seasonal wetland availability for breeding montane amphibian populations (Corn and Muths, 2002; Pilliod et al., 2002). Dependent on winter snowpack levels, spring and summer snowmelt is expected to affect the hydrologic and vegetative conditions necessary for amphibian productivity and dispersal success among wetlands. Is it also expected that precipitation-driven snowmelt variation may alter the spatial configuration of the wetland habitat (Corn, 2005). We therefore consider spatio-temporal snowpack variation as an important potential driver of the hydrological conditions that could influence amphibian genetic connectivity in montane regions.

We predicted genetic connectivity of *P. maculata* among sampled breeding wetlands by within- and between-wetland predictors, as well as wetland connectivity predictors, using gravity models. Gravity models are network models parameterized to include landscape-based attraction and resistance factors to predict genetic distance. Our gravity models also incorporated functional connectivity predictors of species movement in spatially-explicit habitat networks to further predict how landscape spatial heterogeneity affects genetic connectivity. Additionally, we investigated the functional connectivity predictors independently from the within- and between-wetland predictors to evaluate the variation in spatio-temporal wetland structure on the potential connectivity of the wetland network. We hypothesized that spatial heterogeneity in habitat structure and inter-annual variation in snowpack directly control wetland availability in a given year and predict that isolation by distance does not explain amphibian genetic connectivity as well as spatio-temporal precipitation and functional connectivity predictors.

## Methods

### Field collection

Permits for field data and tissue collection were provided by the United States Forest Service and the Colorado Division of Wildlife. All animal procedures in this study followed recommendations of the Colorado State University Institutional Animal Care and Use Committee; this study was approved and permitted by the Colorado Division of Wildlife (# 09HP957) and Colorado State University Institutional Animal Care and Use Committee (# 09107A02).

**Figure 1:**
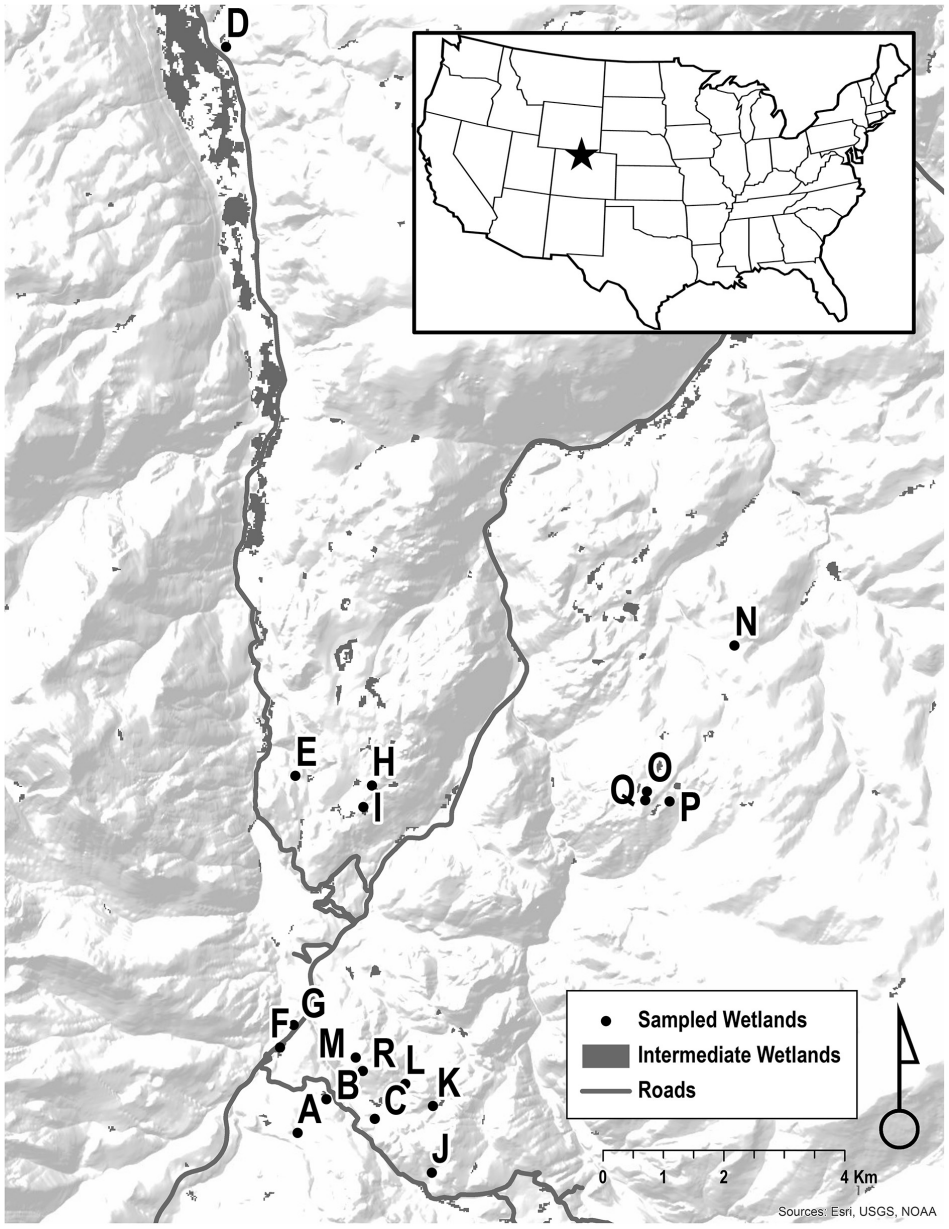
**Study area: Headwaters of the Cache La Poudre River and Laramie River, Colorado, USA**. Sampled breeding wetlands are shown as letters.

We used a stratified random sample design (by elevation) to select sites, augmented by sites used in the Spencer study (Spencer, 1964). Of the 35 surveyed sites in the study area, 22 were occupied in 2009–2010. Eighteen sites yielded a sufficient number of samples to be used in the analysis (*n* = 322 individuals, Table 1). Of the 18 sites with sufficient sample sizes for genetic analysis, 14 were selected via the random stratification (9/14 of these were occupied in Spencer, 1964) while 4 were “augmentation” sites (occupied during the Spencer study but not part of the random stratification; Spencer, 1964; Corn and Muths, 2002). Wetlands varied in area (200–20,000 m^2^) and were visited 1–3 times with the goal of obtaining 30 samples (buccal swabs from adults, Goldberg et al., 2003) or tail clips from larvae (Murphy et al., 2010b; Figure 1). Wetland depths were categorized as < 1 m, 1–2 m, and >2 m. Vegetation cover was estimated by perceived percentage vegetative cover during sampling. pH and conductivity were sampled at each wetland.

**Table 1:** **Boreal chorus frog (***Pseudacris maculata***) sample sizes per studied wetland**.

**Site ID**	**Field ID**	**Name**	**Adults**	**Tadpoles**	**Screened tadpoles**	**Final sample size**
1	3008	Laramie Lake North	9	0	0	9
2	3107	Spruce bog	3	30	24	27
3	3109	Laramie Lake South	11	0	0	11
4	3111	Spencer 7	2	0	0	2
5	3111.2	Old Highway 14	8	19	14	22
6	3112	Sylvatica	29	40	31	60
7	3113	Spencer 16	5	0	0	5
8	3114	Spencer 12	2	30	14	16
9	3114.3	Spencer 11	0	30	27	27
10	3117	Lily	19	0	0	19
11	3117.2	Mosquitos	11	0	0	11
12	3118	Matthews	13	0	0	13
13	3119	Zimmerman 1	0	31	28	28
14	3121.1	Zimmerman 6	0	27	27	27
15	3121.2	Zimmerman 5	0	5	5	5
16	3122.1	Tunnel B	0	30	14	14
17	3124	Lily Pond Lake	2	32	22	24
18	3126	Mosquito 2	2	0	0	2
		Total	116	274	206	322

### Genetic data

DNA was extracted from tissue samples using a Qiagen DNeasy96 tissue kit with minor modifications to manufacturer's protocol (Murphy et al., 2010b). We generated multi-locus genotypes (*n* = 322, loci = 12, Lemmon et al., 2011, Appendix S1) using the Qiagen multiplex kit, an Applied Biosystems 3730 automated sequencer, and scored fragments with Gene Marker 1.91 (SoftGenetics). We implemented a number of measures for quality control: at least 2 negative controls per DNA extraction, 2 negative controls (no DNA) per each PCR amplification, amplified a known genotype in each PCR reaction, re-amplified all rare alleles (< 5% frequency), and re-amplified in at least 10% of samples to assess accuracy of genotyping. When using larvae, varying sample size of full siblings may bias estimates of allele frequencies (Goldberg and Waits, 2009). Therefore, we estimated clusters of full siblings for each sample location (Wang, 2004) and subsampled each sibling cluster (n) where n is the number of individuals in the smallest sibling cluster for that location (Goldberg and Waits, 2009; Murphy et al., 2010b). We tested for Hardy–Weinberg proportions and gametic phase disequilibrium and estimated genetic distance using D_ps_ (Bowcock et al., 1994) with Microsatellite Analyser (MSA) (Dieringer and Schlötterer, 2003) measured for all pairwise comparisons.

### Gravity models

Gravity models (Fotheringham and O'Kelly, 1989) are network-based models that incorporate landscape data that potentially influence genetic connectivity, and factors potentially influencing amphibian population dynamics. In this framework, functional connectivity (*T*_*ij*_, 1-genetic distance, dependent variable) is modeled as a response of three type of independent variables: spatial distribution (distance between sites, *w*), at-site (network nodes, *v*) characteristics representing production of flow and between-site (network edges, *c*) characteristics describing resistance to flow (Equation 1; Murphy et al., 2010a, 2015). Parameter estimates for independent variables are μ, α, and β respectively, where α and β may represent a vector of variables (Anderson, 1979; Fotheringham and O'Kelly, 1989).

(1) $$T_{ij}=kv_{i}^{\mu }w_{j}^{\alpha }c_{ij}^{-\beta }$$

Geographic distance was included in all gravity models as gravity models (“spatial interaction models”) assume spatial autocorrelation (Anderson, 1979). To fit the gravity models as singly constrained, we linearized the equation by taking the natural log and fit using mixed effects models (Murphy et al., 2010a) where each site has an independent estimated intercept (*k*) but global estimate of all independent variables (Murphy et al., 2010a). Singly constrained gravity models balance information content and effective sample size (Fotheringham and O'Kelly, 1989); they also account for non-independence of pair-wise genetic distances (Murphy et al., 2015).

To include spatially-explicit measures of functional connectivity, we included three sets of predictors: (a) within-habitat (*v*) and (b) between-wetland environmental predictors (*c*), and by (c) spatio-temporal wetland connectivity predictors (*c*). Our spatio-temporal wetland connectivity predictors are quantified using three wetland networks: (1) a spatial network, to evaluate connectivity of 18 occupied wetlands; (2) a temporal network, to evaluate effects of annual fluctuations in hydroperiod; and (3) a stepping-stone network, to evaluate the effect of all 128 potential wetlands in the study area on genetic connectivity. All candidate models within 2 ΔAIC (Akaike's information criterion, AIC; Akaike, 1974; Burnham and Anderson, 2002) were considered the top models of connectivity. A null model of isolation by distance (distance alone) was used as a baseline for comparison. Model validation techniques for gravity models are limited. However, our goal was not to infer but to evaluate the relative contribution of within- and between-wetland versus wetlands connectivity predictors over space and time in predicting genetic distance. Accordingly, we included ΔAIC values per single predictor in the top six models as a proxy of predictor contribution to top-ranking gravity models.

#### Within- and between-site predictors

Wetland characteristics are expected to control the number of potential migrants. Therefore, we included within-wetland (node) variables potentially important for recruitment: peripheral habitat (ratio of meadow to forest), run-off (impervious surfaces), water accumulation potential (compound topographic index), precipitation timing (precipitation ratio), site accessibility (relative slope position), and conductivity (see Table 2 for description and justification of variables). All within-wetland characteristics were measured within a 100 m buffer surrounding wetland edge. Between-wetland (edge between nodes) variables are those that are hypothesized to promote or resist dispersal: habitat (ratio of meadow to forest), roads (impervious surfaces), water accumulation potential (compound topographic index), precipitation timing (precipitation ratio), and topographic complexity (surface relief ratio; see Table 2). We tested for correlations among these variables and did not include any variables with an *R*^2^ > 0.7. We found no significant collinearity between remaining predictors using a VIF threshold of 5. Between-wetland variables were evaluated for the 18 primary wetlands using a saturated network (i.e., each wetland is connected to all other wetlands) where the sensitivity of land cover types between wetlands was analyzed at multiple spatial scales, measured as bandwidths along each network edge [30 (minimum resolution), 60, 120, and 240 m buffers, Murphy et al., 2010a,b]. Selection of the best bandwidth to use was evaluated using AIC.

**Table 2:** **Within-wetland, between-wetland, and wetland connectivity predictors**.

**Metric by process**	**Predictor**	**Abbreviation**	**Calculation**	**Description/Justification**	**Res. (m^2^)**	**Source**
Topographic distance	Distance	dist	Topographically-corrected vector length	*P. maculata* are dispersal limited (Spencer, 1964)	10	NED (Gesch et al., 2002)
Within-wetland variables (Production)	Meadow:Forest	M:F_at	Ratio of meadow to forest cells within 100 m of wetland	Meadow habitats have greater water temperature and productivity compared to forest (Pilliod et al., 2002)	1	National Agricultural Imagery Program (2010)
	Impervious surfaces	imperv_at	Count of impervious cells within 100 m of wetland	Runoff and pollution may limit larval development (Sanzo and Hecnar, 2006; Snodgrass et al., 2008)	30	NLCD (Fry et al., 2011)
	Compound topographic index	cti_at	Flow accumulation by catchment size (Moore et al., 1993)	Water holding capacity (Gomez-Rodriguez et al., 2009) is related to hydroperiod	30	SRTM (Jarvis et al., 2008)
	Precipitation Ratio	pratio_at	Ratio of summer precipitation to total precipitation (Rehfeldt et al., 2006)	Summer snowpack melt is important for wetland persistence and amphibian breeding (Corn, 2003)	30	Rehfeldt et al., 2006
	Relative slope position	rsp_at	Position between valley (0) and ridge (1) (Murphy et al., 2010b)	Wetland slope position may deter dispersal and could limit gene flow (Giordano et al., 2007).	30	NED (Gesch et al., 2002)
	Conductivity	EC_at	Field measurement (Murphy et al., 2010a)	Affects embryo survival (Brand et al., 2010) and abundance (Browne et al., 2009)	NA	Field collected
Between-wetland variables (Resistance)	Meadow:Forest	M:F_bet	Ratio of meadow to forest cells	Moisture promotes dispersal (Munger et al., 1998); forests are relatively dry (Goldberg and Waits, 2010)	1	National Agricultural Imagery Program (2010)
	Impervious surfaces	imperv_bet	Mean value of built, impervious land cover (Xian et al., 2011)	Roads may limit amphibian dispersal (Mazerolle, 2004; Arens et al., 2007)	30	NLCD (Fry et al., 2011)
	Compound topographic index	cti_bet	Mean flow accumulation by catchment size (Moore et al., 1993)	Wetness may increase dispersal because of decreased desiccation potential (Bartelt and Peterson, 2005)	30	SRTM (Jarvis et al., 2008)
	Precipitation ratio	pratio_bet	Mean ratio of summer precipitation to total precipitation (Rehfeldt et al., 2006)	Wetness may increase dispersal because of decreased desiccation potential (Murphy et al., 2010b)	30	Rehfeldt et al., 2006
	Surface relief ratio	srr_bet	Mean geometric surface texture in a continuous raster surface (Evans, 1972)	Ridges are often barriers for amphibian dispersal (Funk et al., 2005)	30	NED (Gesch et al., 2002)
Wetland connectivity	Probability of wetland connectivity (Saura and Rubio, 2010): spatial-breeding, temporal-breeding, and stepping-stone networks	PC	Probability (%) that a given wetland contributes to habitat connectivity/availability (sum of Intra, Flux, and Connector, described below)	Amphibian populations often exist in a metapopulation where larger, spatially clustered wetlands are more likely to be recolonized than isolated wetlands (Driscoll, 1997; Rozenfeld et al., 2008; Saura and Rubio, 2010)	–	NED (Gesch et al., 2002); NLCD (Fry et al., 2011)
		Intra	Contribution to connectivity by a given wetland by area of available habitat	Wetland area increases the chance a wetland will be encountered (Hanski and Ovaskainen, 2000)	–	
		Flux	Area-weighted contribution to connectivity by a given wetland by position in the network.	Both spatial position and wetland area contribute to dispersal through a given wetland relative to other wetlands, facilitating functional connectivity of dispersal-limited organisms like many amphibians (Driscoll, 1997)	–	
		Connector	Contribution to connectivity by a given wetland in the network by spatial position alone.	Some wetlands, regardless of area, can facilitate dispersal among wetlands by highly adjacent spatial position relative to other wetlands, influencing genetic connectivity (Fortuna et al., 2006)	–	

Explanatory parameters used to explain genetic distance (D_ps_) included in gravity model analyses and to measure the effect of climate fluctuation scenarios. For wetland connectivity metrics, the same four metrics were calculated for each of the spatial, temporal, and stepping-stone networks.

#### Wetland connectivity predictors

We incorporated wetland connectivity predictors in the gravity models that represented wetland composition and configuration data (i.e., wetland area, spatial position in landscape). These functional connectivity metrics quantify the functional capacity of the landscape to inhibit or facilitate movement and, consequentially, gene flow (Taylor et al., 1993). Functional connectivity predictors were calculated per wetland based on three types of network: (a) a spatial breeding network (“spatial-breeding”) measuring static functional connectivity of 18 breeding wetlands; (b) a temporal network (“temporal-breeding”), measuring fluctuating connectivity per breeding wetland over time, dependent on snowpack variation; and (c) a spatial wetland network (“stepping-stone”) measuring per wetland connectivity considering the sum of 110 additional suitable stepping-stone habitat between 18 breeding wetlands (LinkageMapper v 0.9, McRae and Kavanagh, 2011) in ArcGIS 10.0 (ESRI, 2011).

##### Spatial-breeding network

We calculated functional connectivity of 18 breeding wetlands for one static sampling period using the spatial-breeding network to incorporate the effect of spatially-explicit habitat structure on genetic connectivity in the gravity models. We developed the network using frog-occupied wetlands as graph nodes and Euclidean distance between wetlands as graph edges. For each wetland, we evaluated the probability of connectivity (Saura and Rubio, 2010, Appendix S2), quantified as four per-wetland connectivity metrics: PC (overall), composed of the sum of the three sub-metrics: Intra (probability of wetland connectivity calculated by habitat area alone), Flux (probability wetland connectivity calculated by spatial position, weighted by area), and Connector (probability of wetland connectivity calculated by spatial position alone, Table 2). These metrics were constrained by a dispersal kernel, calculated using the maximum observed dispersal distance of *P. maculata* (~600 m, Spencer, 1964), but assuming that some individuals have the capacity to surpass this maximum distance (5% of individuals).

##### Temporal-breeding network

To evaluate the effect of interannual variation in wetland hydroperiod on *P. maculata* genetic connectivity, we calculated functional connectivity metrics for 18 breeding wetlands over time using the temporal-breeding network. For the temporal-breeding network, using the same frog-occupied wetlands as the spatial-breeding network, we modified the “availability” property of each wetland according to the amount of snowpack (SNOTEL 1979-2010 Station CO05J37S, National Water and Climate Center). We classified observed wetland permanence (Amburgey et al., 2014), a qualitative proxy of ability for wetlands to maintain a suitable hydroperiod for amphibian productivity, under varying snowpack depths. We used the average snotel snowpack depth over recorded years (average depth = 63.5 cm) as a median threshold for wetland permanence (Low permanence < 63.5 cm snow; High permanence > 63.5 cm snow; Neutral = no differences related to snowpack). We designed this novel, qualitative method to classify what conditions were most productive for chorus frogs based on snowpack for a given site: (1) wetlands that are productive only when snowpack is low and breeding areas are available due to decreased water depth (Low); (2) wetlands that are productive only when snowpack is high and breeding areas are available due to increased water depth (High); and (2) wetlands where production is not influenced by the amount of water from snowpack (Neutral). We then attributed the sum of years of available hydroperiod per snowpack category occurring from 1979–2010: “low” wetlands productive 15 of 33 years, “high” wetlands productive 18 of 33 years, and “neutral” wetlands productive in all 33 years. Temporal functional connectivity predictors were quantified using probability of connectivity metrics, as described for the spatial network.

##### Stepping-stone network

To evaluate the effect of neighboring wetlands on *P. maculata* genetic connectivity we included all potential breeding sites (110 additional wetlands, National Land Cover Dataset, 30 m resolution, Fry et al., 2011) to represent nodes in the spatial-breeding network. Only wetlands greater in area than the smallest neutral breeding wetland (>400 m^2^). The resulting stepping-stone network is the sum of the 18 sampled breeding wetlands and 110 wetlands located within the study region and were considered potentially suitable for intermediate habitat, for 128 nodes. We considered all selected wetlands to be neutral to snowpack variation for the stepping-stone network. We calculated functional connectivity predictors as described in the spatial-breeding network only for our 18 sampled, occupied wetlands to quantify the effect of neighboring wetland habitat on functional connectivity on our focal sites.

## Results

### Genetic data

All 11 microsatellite loci were polymorphic, with between 7 and 26 alleles per locus (× = 14.16) and heterozygosity from 0.212 to 0.788 (× = 0.483) by locus (Table S1). Likely due to substructure, loci were not in global Hardy–Weinberg equilibrium (HWE) or linkage equilibrium. However, when considered on a pond-by-pond basis, no single locus was consistently out of HWE or LD, indicating that deviations are unlikely to be due to null alleles. Global *G*_ST_ over all loci was 0.215 and was highly significant. Pair-wise genetic differentiation metrics consistently revealed generally significant levels of genetic differentiation with 65% of pairwise *G*_ST_ comparisons significant after Bonferroni correction (*p*-value 0.05), 84% of non-significant values were sites with less than 700 meters separation. Pairwise *G*_ST_ values ranged from 0 to 0.370. D_ps_ also indicated genetic structure in our study area, ranging from 0.312 to 0.943 (× = 0.553).

### Gravity models

The 30 m bandwidth of between-wetland factors had the lowest competing AIC scores and thus was used for all gravity analyses. The model set with the most support (8 models ΔAIC values < 2) included distance between sites (distance) with within-site moisture (pratio, cti) and between-site resistance (topography as measure by srr, pratio; Table 3, Appendix S3). Models including surface relief ratio within-wetland or precipitation ratio within-wetland as independent predictors resulted in ΔAIC values < 2 but were not the top-ranking models. Functional connectivity predictors from the stepping-stone network (i.e., stepping-stone wetland connectivity) were also important predictors in explaining the genetic connectivity among *P. maculata*-occupied wetlands (PC_steppingstone_at, ΔAIC < 2; Table 3) though did not explain genetic connectivity as well independently (Table 3). Some measures of wetness within-wetland (cti or pratio) were included in six of the eight top models. Surface relief ratio was relevant in all of the competing models while moisture (pratio) was the only other metric describing differences between-wetlands that was present in the top models (three of the eight models). Distance alone (null hypothesis) was not in the top set of models (Table 3).

**Table 3:** **Gravity models that best explain genetic distance**.

**Variables**	**Number of parameters**	**ΔAIC**	**LogLik**
distance, srr_bet, pratio_at, PC_stepping-stone_at	4	0	−6.04
distance, srr_bet, pratio_at, pratio_bet, PC_stepping-stone_at	5	0.70	−5.39
distance, srr_bet, pratio_at, cti_at, PC_stepping-stone_at	5	0.92	−5.51
distance, srr_bet, pratio_bet, pratio_at, cti_at, PC_stepping-stone_at	6	1.62	−4.85
distance, pratio_bet, pratio_at, PC_stepping-stone_at	4	1.70	−6.89
srr_bet	1	1.81	−9.47
pratio_at	1	1.92	−8.20
distance	1	2.73	−9.47
pratio_bet	1	2.96	−8.59
cti_at	1	3.70	−6.96
PC_stepping-stone_at	1	9.33	−11.77

Distance was included in all models, ΔAIC, and log likelihood scores for competing gravity models explaining genetic distance as a proxy for genetic connectivity. Single predictors included in top-ranking models were added as a proxy for relative contribution of individual variables to top-ranking models. The dashed line indicates any model that was not within the threshold of ΔAIC < 2 and therefore not a top model describing genetic connectivity. For abbreviations, see Table 2.

### Wetland connectivity

**Figure 2:**
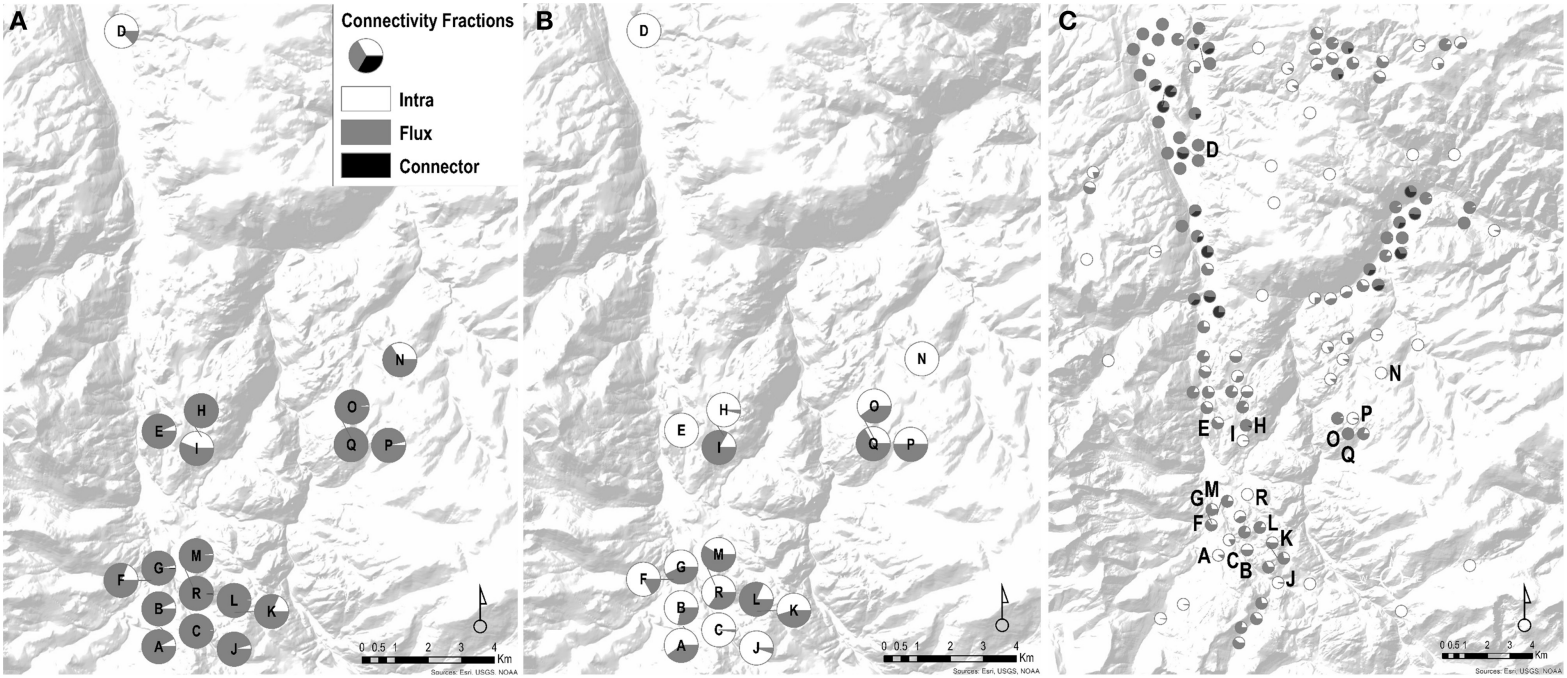
**Networks designed for wetland connectivity of ***Pseudacris maculata*****. Pie charts represent the proportion of influence by Intra, Flux, and Connector to overall wetland connectivity. **(A)** Spatial-breeding network: all sampled sites were considered nodes. In this scenario, wetland connectivity is largely driven by Flux (availability + spatial position). **(B)** Temporal-breeding network: all sampled sites were considered nodes, but categorized as temporal_low, temporal_high, and temporal_neutral wetlands. Compared to the Spatial_breeding network, the temporal network is now driven by both Intra (availability) and Flux (availability + spatial position). **(C)** Stepping-stone network: 110 unoccupied sites within the region were added to the 18 sampled sites. In the stepping_stone network, the Connector fraction now becomes a driver of connectivity for sampled wetlands.

Considering only wetland connectivity metrics in predicting genetic distance, we found that the presence of stepping-stone wetlands (stepping-stone network) was the highest contributor to the top models explaining genetic connectivity (Table 3). Percent-values of wetland connectivity measured using the spatial-breeding, temporal-breeding, and stepping-stone networks were variable perwetland (0.29–45.25%; 0.00–68.47%; 0.00–0.41%, respectively) (Table 4). For the spatial-breeding network, wetland area and wetland spatial position in the network (connectivity characteristics represented by the Flux connectivity metric) contributed most to wetland connectivity (Table 4, Figure 2A) whereby site “I” represented the most functionally connected wetland spatially (*PC* = 45.24%; Figure 2A) due to wetland availability alone (represented by the Intra metric). Conversely, for the temporal-breeding network, only wetland availability (measured by the Intra connectivity metric) contributed to overall wetland connectivity (Figure 2B) whereby site “C” represented the most functionally-connected wetland over time (*PC* = 68.47%). In the stepping-stone network, functional connectivity quantities (*PC*-values) for all 18 sampled sites were low (relatively low Flux, Intra and connection values) but variable. The Connector metric did not contribute to overall wetland connectivity (*PC* < 0.01%) for any wetlands in any of the three types of networks.

**Table 4:** **Functional connectivity, quantified as probability of connectivity, per wetland**.

**Wetland**			**PC (%)**			**Intra (%)**			**Flux (%)**			**Connector (%)**		
**ID**	**Snowpack**	**Area (m^2^)**	**Spatial**	**Temporal**	**Step**	**Spatial**	**Temporal**	**Step**	**Spatial**	**Temporal**	**Step**	**Spatial**	**Temporal**	**Step**
A	Neutral	400	10.3	0.05	2.09E+00	0.8	0.03	1.93E+00	9.5	0.02	1.58E−01	0	0	0
B		3000	11.5	2.1	2.18E+00	0.8	1.51	1.93E+00	10.68	0.59	2.49E−01	2.18E−02	0	0.0004236
C		20000	2.15	68.47	1.05E−01	0.03	67.14	6.79E−02	2.11	1.32	3.73E−02	1.72E−02	0	2.36E−05
D		10000	8.28	16.79	1.74E+01	7.19	16.79	1.74E+01	1.09	0	0.00E+00	0	0	0
E	Low	1800	7.36	0.16	1.14E+00	0.45	0.16	1.09E+00	6.91	0	5.28E−02	1.53E−03	0	0
F		4000	26.52	0.96	1.29E+01	4.99	0.8	1.21E+01	21.52	0.16	8.13E−01	4.45E−03	6.69E−05	3.16E−05
G		2000	5.51	0.35	1.14E+00	0.16	0.2	3.91E−01	5.28	0.15	7.48E−01	6.93E−02	0	0
H	High	8750	1.2	2.75	4.70E−01	0.01	2.66	1.93E−02	1.19	0.1	4.51E−01	0	0	0
I		675	45.24	0.11	4.88E+01	19.97	0.02	4.83E+01	25.25	0.1	5.02E−01	1.66E−02	0	0
J		4000	4.49	0.58	5.09E−01	0.2	0.55	4.83E−01	4.29	0.03	2.62E−02	0	0	0
K		1350	21.07	0.12	9.40E+00	3.82	0.06	9.25E+00	17.23	0.05	1.56E−01	1.22E−02	0	0
L		750	1.98	0.11	1.72E−01	0.02	0.02	5.50E−02	1.94	0.09	1.17E−01	2.00E−02	2.09E−04	3.55E−04
M		6300	4.13	3.34	3.14E−01	0.09	1.38	2.20E−01	4.02	1.96	9.38E−02	1.37E−02	0	1.19E−05
N		700	5.84	0.02	4.79E+00	1.98	0.02	4.79E+00	3.86	0	3.20E−06	0	0	0
O		1000	1	0.05	1.03E−01	0.02	0.03	5.92E−02	0.97	0.02	4.39E−02	3.25E−03	0	0
P		200	1.34	0	1.54E−01	0.05	0	1.21E−01	1.29	0	3.32E−02	0	0	0
Q		12000	0.29	0.02	3.03E−02	0	0.01	4.83E−03	0.29	0.02	2.54E−02	2.96E−03	1.85E−04	0
R		450	1.4	7.67	9.40E−02	0.01	4.99	2.45E−02	1.37	2.67	6.94E−02	2.29E−02	0	1.50E−04

Total connectivity (PC) is the sum of Intra, Flux, and Connector metrics. Spatial, spatial-breeding network; Temporal, temporal-breeding network; Step, stepping-stone network. Neutral, Low, and High snowpack categories refers to wetlands with productivity during all years, low-snowpack years, and high-snowpack years, respectively. Corresponding values are depicted in Figure 2.

## Discussion

Understanding how spatio-temporal variation in habitat composition and configuration influences species dispersal, colonization success, and gene flow is critical to predicting species demographic dynamics in changing landscapes. Variability in the state of environmental conditions on which many species depend may have significant consequences on individual development, population dynamics, and genetic diversity (Funk et al., 2005; Fortuna et al., 2006; Gamble et al., 2007). Considering future climatic and land cover changes, it is essential we understand how fluctuations in environmental factors affect species genetic connectivity toward prediction of demographic and genetic shifts. We compared these environmental factors to elements of functional connectivity that influenced dispersal and gene flow in a population of *P. maculata* over space and time, including climatic fluctuations to address the potential impact of climate warming on the genetic structure of populations. We found that complex interactions among covariates (i.e., within- and between-site moisture, between-wetland topographic complexity, underlying wetland connectivity and fluctuations in annual precipitation), have distinct and potentially critical roles in controlling genetic connectivity in boreal chorus frogs.

### Within-wetland factors

Within-wetland characteristics were important in determining genetic connectivity. Our results indicate stronger genetic connectivity among wetlands with higher levels of water recharge (measured by surface relief ratio and precipitation ratio), a greater capacity to hold water (measured as compound topographic index), and presence in all years (measured as neutral snowpack). Indeed, based on ΔAIC model results, surface relief and precipitation were moisture predictors that explained genetic connectivity independently better than other individual predictors (Table 3). These moisture predictors are related to hydroperiod duration indicating that resources are available for breeding, growth, and development (Gomez-Rodriguez et al., 2009). In an ephemeral habitat, the longer the hydroperiod, the higher the probability that offspring and thus potential migrants will be produced contributing to a greater probability of gene flow (Husband and Barrett, 2002). We therefore suggest that fluctuations in precipitation (snow) directly affect variability in wetland availability and indirectly affect the conditions necessary for frog productivity and dispersal (Driscoll, 1997; Schwartz and Jenkins, 2000). We also suggest that precipitation flux affects between-wetland conditions (i.e., topographic roughness) which could confound dispersal costs (Funk et al., 2005; Semlitsch, 2008) and affect gene flow. Thus, frog populations that are faced with annual variability in precipitation both within- and between wetlands may rely on asynchronous dispersal dynamics to maintain genetic diversity.

### Between-wetland factors

Two between-wetland characteristics influenced *P. maculata* genetic connectivity: topography and moisture. Habitat and landscape controls on functional connectivity are crucial factors that facilitate species persistence and genetic diversity (Funk et al., 2005). In a montane region with relatively high levels of topographic roughness, amphibians are particularly susceptible to genetic isolation (Gomez-Rodriguez et al., 2009; Murphy et al., 2010b). Dispersal between wetlands is facilitated by more moisture suggesting that desiccation risk is an important limiting factor to dispersal. Therefore, topographic roughness inhibits amphibian movement while high-moisture landscape matrix—controlled by snowmelt—facilitates movement for this dispersal-limited species.

The stepping-stone wetland connectivity metrics were considered most important in explaining genetic connectivity despite a much lower magnitude in values relative to the spatial-breeding and temporal-breeding networks. While Euclidean distance alone was not a significant predicator in any of the best models, the presence of wetland habitat between the 18 occupied wetlands was a significant factor. Therefore, wetland spatial heterogeneity likely interacts with fluctuating environmental characteristics to affect amphibian genetic connectivity. Stepping-stone habitat improves a given habitat's probability of connectivity regardless of that habitat's area (Saura and Rubio, 2010). Considering dispersal limitations of *P. maculata*, the presence of stepping stone wetlands possibly improves the probability of dispersal success and gene flow among occupied wetlands and is likely important when there is high topographic between-wetland resistance. This result demonstrates the importance of the underlying habitat spatial heterogeneity compared to fluctuations of critical environmental factors potentially influencing population dynamics of a dispersal-limited species.

### Climatic fluctuation and genetic connectivity

Our results indicate that winter snowfall is essential to the amount and quality of wetland that is available to *P. maculata* and that high snowpack results in consistent inter-annual habitat availability for frogs. Thus, high snowpack is associated positively with gene flow. Because chorus frogs produce large numbers of propagules and show little parental investment, more wetland should facilitate greater production and increased colonization success (Corn, 2005), especially if wetter between-wetland matrix is also available. Conversely, low snowpack might result in fewer available wetlands and fewer stepping-stone wetlands. Thus, low snowpack scenarios may reflect decreased habitat availability, reduced reproduction, decreased colonization and less gene flow. Global models of climate change predict changes in precipitation, both in frequency and amount, and are suggested to impact montane species dramatically (Corn, 2005; Castillo et al., 2014).

### Metapopulation dynamics

We argue that fluctuating environmental conditions in heterogeneous landscapes have a potential role in structuring spatially-explicit populations, and could be important drivers of metapopulation dynamics. Theoretically, a classic metapopulation structure is defined by interbreeding subpopulations linked by dispersal and extinction-colonization dynamics (Smith and Green, 2005). As metapopulations are dynamic, they are influenced strongly by complex and interacting landscape characteristics that affect reproduction and dispersal capacity of individuals (Hanski and Ovaskainen, 2000). Based on the results of our study, we suggest that dramatic changes in available habitat and resistance between habitat patches (i.e., wetlands) influence the functional connectivity of a metapopulation where dispersal is limited, likely controlling genetic connectivity among amphibian subpopulations. Twenty-two of the total 35 sites in the sampling region were occupied in 2009–2010 (we analyzed only 18 in this study due to sample size limitations). Thirteen of the 22 occupied sites in this study were also occupied in an earlier study (21 total occupied sites, Spencer, 1964). However, we found frogs at nine additional sites that were described as lacking frogs in the earlier study (Spencer, 1964) and did not find frogs in eight of the sites described as occupied by frogs in the earlier study. Notably, some of the original occupied sites sampled in the 1964 study were no longer holding water or were considered unsuitable in 2009–2010. These observations suggest that shifts in occupancy have taken place in this landscape over the past 40+ years, likely due to succession and fluctuating snowpack patterns affecting the hydroperiod and availability of suitable within- and between-wetland habitat, similar to changes observed in amphibian habitats elsewhere (McMenamin et al., 2008; Hossack et al., 2015). Annual variability in precipitation (timing and amount) influences a spatially-explicit metapopulation structure (Hanski, 2001) because temporal fluctuations in biotic and abiotic factors can modify the availability of population sources and sinks over space and time (Consentino et al., 2012). If annual variability in precipitation (i.e., snowpack) has significant control over habitat availability, then asynchronous dynamics among subpopulations may be required to consistently recolonize wetlands. We expect this effect because improved connectivity among wetlands increases the ability of individuals to disperse and promotes genetic diversity within the metapopulation.

The effects of environmental controls on metapopulation dynamics are not limited to amphibians (Johst et al., 2002). For example, many turtle species are dispersal-limited and exhibit metapopulation structure (Souza et al., 2002), thus, environmental circumstances may be influencing their genetics. Similarly, fragmented forests affect patch colonization and metapopulation dynamics differently for three mammalian species depending on the species dispersal ability (Lawes et al., 2000), likely influencing genetic connectivity among subpopulations. Finally, African butterflies of the genus *Bicyclus* expressed coupled genetic and physiological plasticity in seasonally-fluctuating environments (Brakefield, 1997). In general, organismal dispersal capability seems to have an important role in population persistence and gene flow within fluctuating, dynamic landscapes and merits future research in landscape genetics.

## Conclusions

Our study used landscape genetic and graph-theoretic connectivity methods to examine how interactions between habitat spatial heterogeneity and climatic variability can influence metapopulation dynamics. The inclusion of fluctuating habitat conditions on species dispersal, colonization, and genetic rescue effects is an essential contribution to advance our understanding of metapopulation ecology. Our work emphasizes the importance in expanding investigations of genetic signatures of populations in dynamic landscapes for multiple species, metapopulations, and metacommunities. Specifically, in a conservation context, these results hold considerable importance in predicting species future responses to human driven land-use and climate change. Considering the likelihood of future climate-driven shifts in precipitation, we expect that fluctuations in habitat availability will continue to affect the metapopulation capacity of *P. maculata* and other dispersal-limited pond-breeding species. We also expect that similar fluctuations may be observed in different landscapes. Empirical investigations of agricultural or urban environments where land use and climate changes may co-occur, such as other montane (Koscinski et al., 2009), agricultural (Youngquist and Boone, 2014), or urban (Hamer and Parris, 2011) landscapes, could be instructional in understanding how metapopulation dynamics are influenced in the face of modified habitat conditions. Reserve design strategies intended to maintain metapopulation persistence should consider underlying habitat spatial heterogeneity together with environmental conditions that influence the dispersal and genetic rescue of dispersal-limited species. Further, temporal data is an essential counterpart to addressing asynchronous metapopulation dynamics and may be the key driver to evaluating species persistence in future ephemeral landscapes. Thus, combined landscape genetic and graph-theoretic approaches to metapopulation ecology will help achieve a more holistic understanding of the complex landscape-climate interactions and species population persistence under dramatic environmental change.

## Author contributions

Conceived and designed the field work: MM, EM, WF. Performed the experiments: MM. Analyzed the genetic data: SB, BJ, SM, MM. Gravity modeling: MM, PS. Wetland connectivity: MF, AW. Wrote the paper: AW, PS, EM, MM. All authors provided substantial review and comments to written manuscript.

### Conflict of interest statement

The authors declare that the research was conducted in the absence of any commercial or financial relationships that could be construed as a potential conflict of interest.
