# Supplementary material for: How spatio-temporal habitat connectivity affects amphibian genetic structure
Source: Front Genet. 2015 Sep 8;6:275. doi: 10.3389/fgene.2015.00275 (PMC4561841; doi:10.3389/fgene.2015.00275)
Supplement: Supplementary file 1 [file DataSheet1.DOCX]

**Appendix S1. Microsatellite Primers**

| *Name* | *Primer sequences* | *Repeat type* | *AT* | *Size Range* | *Label* | *H_0_* |
| --- | --- | --- | --- | --- | --- | --- |
| A8 | (Lemmon et al 2011) | (CA)_n_CG(CA)n | 60 | 131-163 | 6FAM | 0.438 |
| D_D11b | (Lemmon et al 2011) | (TCTA)_n_ | 60 | 344-436 | NED | 0.788 |
| D_E10 | (Lemmon et al 2011) | (AC)nA(TATC)nTAA(TATC)n | 60 | 380-432 | 6FAM | 0.449 |
|  |  | TCA(TATC)nTAA(TATC)n |  |  |  |  |
|  |  | TCA(TATC)nTAA(TATC)nTCA(TATC)n |  |  |  |  |
| P05^1^ | F – CTGATGGCAGCCCTGAAC | (AC)_n_TT(AC)_n_ | 60 | 128-167 | VIC | 0.337 |
|  | R - GGAAATTTAAAGCCACATCAGG |  |  |  |  |  |
| P08^1^ | F – TTGCTGTGAATGCAATTTTGG | (AC)_n_AA(AC)_n_ | 57.5 | 128-167 | PET | 0.7 |
|  | R – GCCCCAAAACTGTGTAATCTG |  |  |  |  |  |
| P12^1^ | F – GGCAGAGCAGCTGTAAGGAG | (GT)_n_ | 57.5 | 79-140 | VIC | 0.402 |
|  | R - CCAGCCCTGCCTATCTTTG |  |  |  |  |  |
| P13^1^ | F – AGGATTTGTGCAATGAGGATG | (CA)_n_CG(CA)_n_ | 60 | 129-167 | PET | 0.61 |
|  | R- CAGGGAAAGAGAGAGCTGGTC |  |  |  |  |  |
| P18^1^ | F- TTTCCGAGCAGACATTACACC | (CT)_n_…(GT)_n_ | 57.5 | 228-276 | VIC | 0.212 |
|  | R- TGGACAGGGAGGAAATAAACAG |  |  |  |  |  |
| P19^1^ | F- GCCCTATGGCCTTTACAG | (AC)_n_ | 57.5 | 55-98 | NED | 0.357 |
|  | R -GATCTGGGGTAGGAATGGTG |  |  |  |  |  |
| P21^1^ | F – ATCCATGTGATTTCCTGCTG | (CA)_n_AA(CA)_n_ | 57.5 | 181-217 | PET | 0.608 |
|  | R – TAGGCTATGTTGCGTTGTGC |  |  |  |  |  |
| P25^1^ | F-TAAGCCGTGGAGACTGGAAG | (CA)_n_ | 57.5 | 91-181 | 6FAM | 0.579 |
|  | R – TTTTCCATCATGGCAGACG |  |  |  |  |  |
| P29^1^ | F-CACCATCTGGGAGGTGCTAC | (GT)_n_ | 57.5 | 152-187 | VIC | 0.311 |
|  | R - ATGTCAAAGCCGACCAAATC |  |  |  |  |  |

^1^Developed by Ecogenics and provided by J. Van Buskirk. Microsatellite loci were identified for *P. maculata* from Isle Royale, MI USA.

**Appendix S2. Probability of connectivity metrics.**

Connectivity per wetland was evaluated by probability (%) of connectivity for the spatial, temporal, and intermediate graphs. This measure is a graph-based metric of habitat availability that quantifies functional connectivity of any node *k* (wetland) (Conefor Sensinode 2.2, Saura and Pascual-Hortal 2010):

*dPC_k_ = dPCintra_k_ + dPCflux_k_ + dPCconnector_k_*

where *dPCintra_k_* (i.e., Intra) represents habitat area provided by wetland *k* , *dPCflux_k_* (i.e., Flux) represents both habitat availability in wetland *k* and its connection to other wetlands in the network, and *dPCconnector_k_* (i.e. Connector) is a measure of connectivity between other wetlands that only represents topological position in the network. In this case, a wetland that is large but topologically isolated in the network may have a large intra value but a poor connector value. We assumed a probability of 5.0 % of all individuals likely surpass the maximum dispersal distance of 600m as recorded by Spencer in 1964.

**Appendix S3. Candidate gravity models to predict genetic connectivity.**

|  | Variables | AIC | ΔAIC | logLik |
| --- | --- | --- | --- | --- |
| 1 | srr_bet, pratio_at, PC_steppingstone_at | 26.08855 | 0 | -6.04428 |
| 2 | srr_bet, pratio_at, pratio_bet, PC_steppingstone_at | 26.78739 | 0.69884 | -5.39369 |
| 3 | srr_bet, pratio_at, cti_at, PC_steppingstone_at | 27.01129 | 0.92274 | -5.50565 |
| 4 | srr_bet, pratio_bet, pratio_at, cti_at, PC_steppingstone_at | 27.7046 | 1.61605 | -4.8523 |
| 5 | pratio_bet, pratio_at, PC_steppingstone_at | 27.78295 | 1.6944 | -6.89147 |
| 6 | Distance Only | 26.95394 | 2.73318 | -9.47697 |
| 7 | srr_bet, pratio_at, PC_Spatial_breeding_at | 30.04629 | 3.95774 | -8.02315 |
| 8 | srr_bet, pratio_bet, pratio_at, PC_Spatial_breeding_at | 30.73577 | 4.64722 | -7.36788 |
| 9 | srr_bet, pratio_at, PC_Temporal_breeding_at | 31.01692 | 4.92837 | -8.50846 |
| 10 | srr_bet, pratio_bet, PC_steppingstone_at | 31.42218 | 5.33363 | -8.71109 |
| 11 | srr_bet, pratio_at, cti_at, PC_Spatial_breeding_at | 31.43549 | 5.34694 | -7.71775 |
| 12 | srr_bet, pratio_bet, PC_Spatial_breeding_at | 31.57247 | 5.48392 | -8.78624 |
| 13 | srr_bet, pratio_bet, pratio_at, PC_Temporal_breeding_at | 31.70082 | 5.61227 | -7.85041 |
| 14 | srr_bet, pratio_bet, PC_Temporal_breeding_at | 31.89409 | 5.80554 | -8.94704 |
| 15 | srr_bet, cti_at, PC_Spatial_breeding_at | 31.99718 | 5.90863 | -8.99859 |
| 16 | srr_bet, cti_at, PC_steppingstone_at | 32.04311 | 5.95456 | -9.02156 |
| 17 | srr_bet, pratio_bet, pratio_at, cti_at, PC_Spatial_breeding_at | 32.08877 | 6.00022 | -7.04439 |
| 18 | srr_bet, pratio_at, cti_at, PC_Temporal_breeding_at | 32.2507 | 6.16215 | -8.12535 |
| 19 | srr_bet, cti_at, PC_Temporal_breeding_at | 32.28823 | 6.19968 | -9.14412 |
| 20 | srr_bet, pratio_at, PC_Spatial_breeding_at | 32.45912 | 6.37057 | -9.22956 |
| 21 | srr_bet, pratio_bet, pratio_at, cti_at, PC_Temporal_breeding_at | 32.89279 | 6.80424 | -7.4464 |
| 22 | PC_Spatial_breeding_at | 33.24058 | 7.15203 | -11.6203 |
| 23 | PC_steppingstone_at | 33.54767 | 7.45912 | -11.7738 |
| 24 | PC_Temporal_breeding_at | 33.64387 | 7.55532 | -11.8219 |
| 25 | pratio_bet, pratio_at, PC_Temporal_breeding_at | 33.65262 | 7.56407 | -9.82631 |
| 26 | srr_bet, pratio_at, PC_Temporal_breeding_at, PC_steppingstone_at, PC_Spatial_breeding_at | 37.77071 | 11.68216 | -9.88536 |
| 27 | srr_bet, pratio_at, PC_Temporal_breeding_at, PC_steppingstone_at, PC_Spatial_breeding_at | 37.77071 | 11.68216 | -9.88536 |
| 28 | srr_bet, pratio_at, pratio_bet, PC_Temporal_breeding_at, PC_steppingstone_at, PC_Spatial_breeding_at | 38.4971 | 12.40855 | -9.24855 |
| 29 | srr_bet, pratio_at, cti_at, PC_Temporal_breeding_at, PC_steppingstone_at, PC_Spatial_breeding_at | 39.80189 | 13.71334 | -9.90094 |
| 30 | PC_Temporal_breeding_at, PC_Spatial_breeding_at | 39.97781 | 13.88926 | -13.9889 |
| 31 | PC_steppingstone_at, PC_Spatial_breeding_at | 40.01034 | 13.92179 | -14.0052 |
| 32 | PC_Temporal_breeding_at, PC_steppingstone_at | 40.40353 | 14.31498 | -14.2018 |
| 33 | srr_bet, pratio_bet, pratio_at, cti_at, PC_Temporal_breeding_at, PC_steppingstone_at, PC_Spatial_breeding_at | 40.50181 | 14.41326 | -9.2509 |
| 34 | pratio_bet, pratio_at, PC_Temporal_breeding_at, PC_steppingstone_at, PC_Spatial_breeding_at | 40.61765 | 14.5291 | -11.3088 |
| 35 | pratio_bet, pratio_at, PC_Temporal_breeding_at, PC_steppingstone_at, PC_Spatial_breeding_at | 40.61765 | 14.5291 | -11.3088 |
| 36 | srr_bet, PC_Temporal_breeding_at, PC_steppingstone_at, PC_Spatial_breeding_at | 44.29606 | 18.20751 | -14.148 |
| 37 | srr_bet, pratio_bet, PC_Temporal_breeding_at, PC_steppingstone_at,PC_Spatial_breeding_at | 44.83742 | 18.74887 | -13.4187 |
| 38 | srr_bet, cti_at, PC_Temporal_breeding_at, PC_steppingstone_at, PC_Spatial_breeding_at | 45.09919 | 19.01064 | -13.5496 |
| 39 | PC_Temporal_breeding_at, PC_steppingstone_at, PC_Spatial_breeding_at | 46.88603 | 20.79748 | -16.443 |
| 40 | cti_bet, imperv_bet, pratio_bet, srr_bet, meadow_bet, cti_at, MF_at, imperv_at, pratio_at, rsp_at, PC_Temporal_breeding_at, PC_steppingstone_at, PC_Spatial_breeding_at | 73.41198 | 47.32343 | -19.706 |
